# MSFE-YOLO: A Steel Surface Defect Detection Algorithm Integrating Multi-Scale Frequency Domain and Defect-Aware Attention

**DOI:** 10.3390/s26082311

**Published:** 2026-04-09

**Authors:** Siqi Su, Jiale Shen, Peiyi Lin, Wanhe Tang, Weijie Zhang, Zhen Chen

**Affiliations:** 1 School of Mechanical and Energy Engineering, Guangdong Ocean University, Yangjiang 529500, China; 18219521228@stu.gdou.edu.cn (S.S.); 15017778995@stu.gdou.edu.cn (J.S.); linpeiyi@gdou.edu.cn (P.L.); tangwh@gdou.edu.cn (W.T.); 43811333vv@stu.gdou.edu.cn (W.Z.); 2 State Key Laboratory of Mechanical Transmission for Advanced Equipment, Chongqing University, Chongqing 400044, China

**Keywords:** multi-scale feature extraction, frequency domain enhancement, attention mechanism, adaptive feature fusion, YOLOv11

## Abstract

Detecting surface defects on steel products is crucial for maintaining quality standards in industrial manufacturing. However, existing detection algorithms face several challenges, including the difficulty of capturing multi-scale defect characteristics with fixed receptive fields, insufficient utilization of defect edge and frequency domain features, and simplistic feature fusion strategies. In response to the above challenges, this paper proposed the Multi-Scale Frequency-Enhanced YOLO (MSFE-YOLO) algorithm that integrates multi-scale frequency domain enhancement with defect-aware attention mechanisms. First, a Multi-Scale Frequency-Enhanced Convolution (MSFC) module was constructed, which extracted multi-scale spatial features in parallel through depth-adaptive dilated convolutions, explicitly modeled high-frequency edge information using the Laplacian operator, and achieved adaptive fusion of multi-branch features via learnable weights. Second, a Cross-Stage Partial with Multi-Scale Defect-Aware Attention (C2MSDA) module was designed, integrating Sobel operator-based edge perception, multi-scale spatial attention, and adaptive channel attention to collaboratively enhance features across spatial, channel, and edge domains through a gated fusion strategy. Finally, an Adaptive Feature Fusion Enhancement (AFFE) module was proposed to achieve adaptive aggregation of multi-level features through a data-driven weight generation network and cross-scale feature interaction mechanism. Experimental results on the NEU-DET and GC10-DET datasets demonstrated that MSFE-YOLO achieved the mAP@0.5 of 79.8% and 66.7%, respectively, which were 1.7% and 2.1% higher than the benchmark model YOLOv11s respectively, while maintaining an inference speed of 89.3 FPS, which satisfied the real-time detection requirements in industrial scenarios.

## 1. Introduction

The steel industry, as the cornerstone of modern manufacturing, plays a crucial role in other industries such as construction, automobiles, and aerospace [1]. According to the World Steel in Figures 2025 released by the World Steel Association on 5 June 2025, the global crude steel production reached 1.885 billion tons in 2024, and the crude steel production of China reached 1.005 billion tons, accounting for 53.3% of the total global output. With the in-depth promotion of Industry 4.0 and intelligent construction strategy, steel enterprises have established more stringent requirements for product quality control. During the steel production process, due to rolling temperature fluctuations, equipment wear, and uneven cooling, various surface defects are inevitable [2]. These defects not only affect the appearance quality of steel products but also reduce their mechanical properties, which may lead to structural failure and major safety accidents [3]. Common surface defects in steel can be broadly categorized into texture-type defects, such as crazing, scratches, and rolled-in scale, and morphology-type defects, including pitted surfaces, patches, and inclusions. These defects exhibit considerable diversity in terms of scale, morphology, and contrast, posing significant challenges to automated detection algorithms. Specifically, defect detection algorithms must simultaneously address multi-scale targets, complex textures, and varying degrees of foreground–background similarity. Therefore, it is extremely urgent to improve the accuracy of steel defect detection.

The traditional defect detection method mainly relies on manual sampling inspection [4]. By randomly sampling samples with the naked eye of the inspectors, there was an imbalance in sampling, which led to large errors in the detection results. Moreover, the detection results were highly dependent on the subjective judgment of the inspectors and were greatly influenced by environmental and personal experience differences. The defect detection method [5] based on machine learning realizes the automatic identification and classification of defects by learning feature patterns from steel sample data, and it has higher detection accuracy and adaptive ability than traditional methods. Liu et al. [6] extracted the surface defect texture features of steel strips using the Local Binary Pattern (LBP) and then input the features into the Extreme Learning Machine (ELM) classifier for recognition, effectively meeting the demand for rapid online defect detection. Zaghdoudi et al. [7] extracted defect features using two texture descriptors, LCCMSP and DCP, and then input the features into Support Vector Machine and Random Forest classifiers, respectively, to construct four basic models. Finally, they integrated the results of multiple classifiers through a Bayesian fusion strategy for defect classification. All the above-mentioned methods used traditional methods for feature extraction and then applied machine learning methods to classify the features. However, in actual steel production, most defects had irregular distribution and indistinct texture features. Therefore, it was difficult to accurately extract features relying solely on traditional feature extraction methods, which led to difficulties in the use of detection algorithms and low detection accuracy and efficiency.

Deep learning methods automatically extract features from input data, not only retaining the advantages of no need for human intervention and avoiding environmental influences but also eliminating the need for manual feature extraction. The single-stage object detection method regards object detection as a regression problem. It directly predicts the bounding box position and category probability from the input image through a single neural network without generating candidate regions, which has the advantages of fast detection speed and simple model structure. To address the difficulties in detecting small targets and the interference of complex background textures in metal surface defect detection, Zhou et al. [8] used the CSPlayer module to replace the original C3 module. By introducing 5 × 5 depthwise convolution and depthwise separable convolution, they enhanced the feature extraction capability while maintaining lightweight. Additionally, the Global Attention Mechanism (GAMAttention) was embedded in the backbone network to enable the model to adaptively focus on target regions while suppressing background interference. Ruan et al. [9] proposed the EPSC-YOLO algorithm, which enhanced multi-scale feature extraction by introducing an Efficient Multi-Scale Attention (EMA) module into the backbone network. Two pyramid convolution modules (PyConv2 and PyConv3) were designed to extract multi-scale features in parallel using convolution kernels of varying sizes, reducing parameter count while improving feature capture capability. A CISBA module integrating channel attention, involution operations, and spatial attention was proposed to enhance local feature representation through dynamically generated convolution kernels.

Although existing studies have achieved significant progress, steel defect detection still faces several challenges. The fixed receptive fields in current defect detection algorithms struggle to capture the multi-scale characteristics of defects [10]. Furthermore, the directional and frequency domain features of defects have not been fully exploited, and the feature fusion strategies remain overly simplistic, neglecting the differentiated contributions of high-level semantic information and low-level detailed information across various detection tasks. Therefore, this paper proposed a steel surface defect detection algorithm based on multi-scale frequency domain and defect-aware attention, termed Multi-Scale Frequency-Enhanced YOLO (MSFE-YOLO). By deeply fusing multi-scale, multi-directional, and multi-frequency features, the proposed method achieved high-precision real-time detection. The main innovations and contributions of this paper were as follows:

(1) Aiming at the problem that the conventional convolution with a fixed receptive field was unable to capture the multi-scale defect features and the lack of frequency domain information modeling, this paper constructed the Multi-Scale Frequency-Enhanced Convolution (MSFC) module. Depth-adaptive dilated convolutions were employed for parallel multi-scale feature extraction, a Laplacian operator was incorporated to explicitly model high-frequency edge information, and an adaptive fusion strategy with learnable weights was utilized, enabling efficient extraction and enhancement of defects across varying scales and fine textures.
(2) In response to the shortcomings of Cross-Stage Partial with Position-Sensitive Attention (C2PSA) in capturing defect edge details and modeling multi-scale features, this paper constructed a Cross-Stage Partial with Multi-Scale Defect-Aware Attention (C2MSDA) module. By introducing an edge-aware module based on Sobel operator, a multi-scale spatial attention mechanism, and an adaptive gating fusion strategy, the collaborative enhancement of spatial domain, channel domain, and edge domain features was achieved, significantly improving the detection accuracy of small-sized and low-contrast defects.
(3) In response to the shortcomings of traditional Concat operations with fixed weights and one-way feature transfer, this paper constructed an Adaptive Feature Fusion Enhancement (AFFE) module. By introducing a data-driven weight generation network, cross-scale feature interaction mechanism, and lightweight fusion strategy, it achieved adaptive aggregation of multi-scale features and cross-level information exchange, significantly improving the detection accuracy and robustness of defects at different scales.

## 2. Related Works

### 2.1. Convolution Module

Convolutional neural networks, as the core technology of deep learning in the field of computer vision, have a direct impact on the quality of feature extraction and the computational efficiency of the model through the design of their convolutional modules. With the continuous growth of industrial intelligence demand, researchers have proposed various innovative convolution structures based on standard convolution for target detection, especially industrial defect detection tasks. These structures significantly reduce computational complexity and parameter count to provide the possibility for model deployment on resource-limited devices.

Conventional convolution, as the fundamental component of convolutional neural networks, extracts features by weighting and summing the local receptive fields of the input feature map. VGGNet [11] demonstrated the importance of network depth for performance improvement by stacking multiple 3 × 3 convolution kernels and achieved significant results on the ImageNet dataset. However, as the number of network layers increases, the disappearance of gradients and the sharp increase in computational complexity become the main factors restricting the deepening of the model. Based on this, ResNet [12] effectively solved the problem of difficulty in training deep networks by introducing residual learning framework and skip connections, making it possible to extend the network depth to 152 layers.

In order to reduce model complexity and improve inference speed, MobileNet [13] first applied depthwise separable convolution to mobile vision tasks. By integrating the conventional convolution into depthwise and pointwise convolution, the computational complexity can be reduced to 1/8–1/9 of the original, while minimizing the loss of accuracy [14]. Dilated convolution expands the receptive field by inserting zero values between convolution kernel elements, capturing a larger range of contextual information without increasing the number of parameters [15]. The Atrous Spatial Pyramid Pooling module employed parallel dilated convolution branches with varying dilation rates to capture multi-scale context, achieving excellent performance in semantic segmentation tasks [16]. The combination of dilated convolution and lightweight architectures provides an solution for real-time defect detection. A lightweight image classifier integrating dilated convolution and depthwise separable convolution has been proposed, which maintains high classification accuracy while reducing computational burden [17].

The fixed sampling grid of the conventional convolution is not effective in modeling geometrically deformed targets. Deformable convolution [18] resolves this issue by also training 2D offsets at each 2D sampling point in order to shape the convolutional kernel of the sampling positions to follow the geometry of the target. For steel surface defect detection, Zhao et al. [19] added deformable convolution to the second and fourth stages of the ResNet-50 backbone and presented Feature Pyramid Network (FPN) to incorporate multi-scale feature fusion that resulted in an mAP of 75.2% on the NEU-DET dataset. Chen et al. [20] proposed DCAM-Net that integrated deformable convolution and attention mechanisms to be able to locate strip steel surface defects fast. The advantages of deformable convolution in modeling complex-shaped defects have established it as a crucial technique in industrial inspection.

The problem of identifying elongated and tortuous defects was overcome by Qi et al. [21], who introduced the concept of dynamic snake convolution that was formulated to work on tubular structure segmentation. This technique can be used to enable the convolutional kernel to meander across the target structure as a snake, avoiding the receptive field drifting into background scores by introducing deformation offsets with continuity constraints. Snake convolution reveals distinctive capabilities in the detection of linear and curvilinear defects in industrial detection. In case of long-marked defects on the steel surfaces, MDSC-YOLOv9 [22] employed multi-scale dynamic snake convolution to enhance feature representation capability and sensitivity for narrow defect patterns. SnakeNet [23] redesigns the neck network using dynamic snake convolution, enhancing the performance at the complex samples and large-scale defects of the model [22]. Snake convolution yields very fine local image features by changing the shape of the kernels dynamically, and, in this process, it has been proven that it has more ability to extract features, and feature capturing has also been shown to be reliable in the case of complex images [24].

The integration through the application of different convolutional structures in the industrial defect detection shows an increasing tendency. Scholars do not only focus on one type of convolution, but they combine and strategically integrate different types of convolutional designs depending on the demands of the detection task. For example, depthwise separable convolution is often used to build lightweight backbone network architecture in applications where resource-constrained edge device is used (such as deployment). Dilated convolution is widely used for capturing multi-scale information during feature fusion. For defect targets with complex shapes, deformable convolution and snake convolution provide geometry-adaptive modeling capabilities. This targeted modular design strategy enables detection models to achieve an optimal balance among accuracy, speed, and computational overhead, providing robust support for the practical deployment of intelligent industrial inspection systems. Similar modular integration strategies have also been validated in improved YOLO architectures for other challenging detection scenarios [25].

### 2.2. Attention Mechanism

Attention mechanisms can be used to simulate the selective attention properties of the human visual system with the neural networks selectively integrating salient information into the input features and inhibiting redundant information to efficiently feature-represent, as well as detect features, thus considerably increasing the capability of feature-representing and detection accuracy [26]. Attention mechanisms have become one of the key methods of enhancing the performance of a model in the object detection case. In the case of defect detection activities in stationary images, channel attention, channel-spatial joint attention, self-attention, and other new attention functions are the main focus of discussion in the present paper concerning the use and development of object detection.

Channel attention mechanisms can learn the weights of the various channels of features to adaptively boost the information-giving channels and reduce the irrelevant channels and thus enhance feature discriminability. The Squeeze-and-Excitation Networks (SENet) [27] is a seminal contribution to channel attention, condensing spatial dimensions with a global average pool layer and utilizing fully connected layers to acquire inter-channel interaction. Nonetheless, its dimensionality reduction operation can also have caused the loss of channel information. Expanding on this, Efficient Channel Attention (ECA) [28] proposed an approach to building a 1D convolution strategy, which enabled the local cross-channel interaction with changing adaptive kernel size without loss of information in dimensionality reduction. In detecting surface defects in steel, Xiang et al. [29] suggested the Adaptive Graph Channel Attention (AGCA) module that is more novel and thus introduces the graph convolution theory to channel attention, where each channel is regarded as a graph node and the adjacency matrices are used to represent topological relationships among the channels.

Even though both channel attention and spatial attention have been successful in enhancing features individually, single-dimensional attention processes find it difficult to fully address the complexity of defect features. The channel-spatial joint attention processes combine the two types of attention in series or parallel arrangement with organic integration of what to attend to and where to attend to. The technique allows both spatial and channel dimension refining of features, which greatly improves the feature representation ability of a network, as well as the accuracy of defect detection. As a representative work, Convolutional Block Attention Module (CBAM) [30] sequentially applied the Channel Attention Module (CAM) and Spatial Attention Module (SAM) to refine feature maps along channel and spatial dimensions, respectively. To deal with the lack of feature extraction capacity and poor feature fusion in the detection of defects on the surface of hot-rolled steel strips, Zhang et al. [31] introduced the multi-scale feature fusion (MSF) strategy, which can improve target defects contents by efficiently fusing both shallow and deep features, getting a higher semantic information without the loss of its resolution. Another thing that they presented is the CSPlayer Res2Attention block (CRA block), which incorporates both Res2Net block and CBAM attention mechanism into the CSPlayer, minimizing the loss of defect information during hierarchical propagation, improving fine-grained feature extraction capacity, and improving the perception of fine features and the overall features.

Surface defects of steel have complicated spatial distributions and long-term interdependences. Self-attention mechanisms bring global contextual information by computing the correlation between any two positions in a sequence, which breaks the shortcomings of local receptive fields of CNNs and offers new technical avenues to the identification of intricate defect patterns. After self-attention mechanisms [32] were effective in natural language processing, Vision Transformer (ViT) [33] brought this new technique to the computer vision domain and was the first system to use pure attention-based architectures in visual tasks. Zhao et al. [34] presented RT-DETR (Real-Time Detection Transformer) in real-time object detection that separated multi-scale features into intra-scale interaction and cross-scale fusion by an effective encoder (hybrid encoder). The use of RT-DETR-R50 with 53.1% AP at 108 FPS on the COCO dataset was the first time that transformer-based architectures had been shown to be better than the YOLO series in real-time detection.

In response to inquiries on the weaknesses of traditional attention mechanisms in given situations, researchers have come up with a number of innovative attention mechanisms in both theories and practical application. Coordinate attention (CA) [35] addressed the problem of losing spatial positional information during global pooling in the conventional channel attention by splitting channel attention into two 1D encoding tasks that the inter-operating feature pools in two spatial dimensions, thereby retaining fine-grained positional information and learning long-range interactions at the same time. Yang et al. [36] also incorporated CA in the DCA-YOLOv8 cattle detection system, which increased the mAP of the system by 13, 4.9, and 15.1 percent over SSD, RetinaNet, and CornerNet respectively. Global Attention Mechanism (GAM) [37] was an information theoretic approach that used the sub-modules of serial channel and spatial attention in an encoder–decoder architecture using a two-layer MLP in order to increase cross-dimensional feature interaction to decrease information redundancy and increase global representation ability. To address the difficulties in detecting small targets and the interference of complex background textures in metal surface defect detection, Zhou et al. [8] used the CSPlayer module to replace the original C3 module. By introducing 5 × 5 depthwise convolution and depthwise separable convolution, they enhanced the feature extraction capability while maintaining a lightweight design. Additionally, Global Attention Mechanism (GAMAttention) was embedded in the backbone network to enable the model to adaptively focus on target regions while suppressing background interference.

With the complexity and diversification of detection targets, the research of attention mechanism in the field of steel defect detection has developed from the application of a single module to the stage of multi mechanism cooperation. The channel attention is the best feature selection, spatial attention is the localization of key regions, self-attention is the global dependency modeling, and new mechanisms such as coordinate attention and global attention are devoted to particular problems, such as positional information storage and feature redundancy. The current research trend indicates that a single attention mechanism often fails to comprehensively address the multiple challenges of diverse scales, complex shapes, and low contrast of surface defects in steel. Therefore, how to reasonably select and combine different types of attention mechanisms based on the inherent properties of defect features while maintaining the model’s lightweight design and achieving collaborative enhancement of multi-dimensional features has become the key to improving detection performance. This development direction lays the theoretical foundation for building a more robust and efficient steel defect detection system. The integration of attention mechanisms with state space models such as Mamba has emerged as a new paradigm, achieving improved trade-offs between long-range dependency modeling and computational efficiency in YOLO-based detection frameworks [38].

### 2.3. Feature Fusion Module

Deep neural networks extract features through convolution and pooling operations, and the shallow features are richly represented in spatial details and positioning. However, they have weak semantic representational ability, whereas deep features have strong semantic abstraction ability but lack serious spatial details loss during downsampling. The challenges faced by surface defect detection of steel lie in the large span of defect scales, complex background textures, and weak features of small-sized defects. Single level features are unable to simultaneously meet the dual requirements of positioning accuracy and semantic understanding. Therefore, effectively integrating features from different levels and enhancing semantic expression while preserving spatial details has become the key to improving detection performance.

Channel concatenation (Concat) is the simplest form of the feature fusion technique that simply layers feature maps of two or more network layers directly along the channel dimension [39]. Mi et al. [40] compared between two fusion strategies—feature map addition (Add) and feature map concatenation (Concat)—in steel strip surface defect detection. The experimental results showed that the Concat operation evaded the loss of feature information and achieved better fusion of multi-level features compared to the Add method, which was better in terms of precision and average precision. But simple Concat functions place the same weight on all input features without any distinction of the various contributions of various feature layers to the representation of targets of different scales. To address the limitations of unidirectional information flow, Liu et al. [41] proposed the Path Aggregation Network (PANet), which provided the path enhancement structure of bottom-up on top of the top-down pathway of FPN. The bidirectional propagation process allowed localization signals of lower layers to propagate up the graph to higher layers more quickly, hence reducing the distance between the top features at lower levels and the top features. Nevertheless, PANet continued to adopt a fixed network topology and even feature weights, and it did not take the differentiated contribution of influences by features in different scales on the final detection results into consideration.

To address the problem of fixed feature weights, Tan et al. [42] proposed the Bidirectional Feature Pyramid Network (BiFPN), which was a key breakthrough toward the transformation of feature fusion as a passive stacking to an active selection. BiFPN has developed an adaptable weighting of different input features, so that the network can intelligently learn fusion weights. At the same time, the network removes the nodes with only a single input and limits the cross-scale fusion in the same layer, which simplifies the network structure while maintaining the interpretability of the model. Zhang et al. [43] proposed the Cross-Scale Weighted Feature Fusion Network (CSWFFN) based on YOLOv5s. The network realized multi-scale feature fusion by embedding the residual module (C3-BiFPN) in the improved BiFPN and enhanced the edge features of defects combined with Laplace sharpening technology. It achieved 86.8% mAP on the NEU-DET dataset and performed well in the detection of small-scale defects. The improved YOLOv9 algorithm proposed by Zhang et al. [44] introduced the BiFPN module, which enhanced the information flow between different scales through the bidirectional feature fusion strategy and also achieved remarkable results in small target defect recognition. These studies have indicated that weighting schemes that are learnable have the ability to achieve greater adaptability to feature fusion. Nevertheless, BiFPN cannot be very flexible to complex situations due to the fixed topology of this model. Regarding the cross-domain application of feature fusion strategies, Zhao et al. [45] proposed the Mussel-YOLO semi-supervised detection framework for mussel detection in underwater acoustic images. The progressive adaptive Feature Pyramid Network (PAFPN) incorporated in this framework achieves progressive fusion between adjacent hierarchical levels through an adaptive weight allocation mechanism, effectively alleviating the information discontinuity caused by direct cross-level fusion. This work validates the effectiveness of adaptive feature fusion strategies in detection tasks with complex backgrounds.

The evolution path of feature fusion technology clearly shows the development context from simple splicing to intelligent aggregation, from fixed weight to adaptive weighting, and from unidirectional propagation to bidirectional interaction. Even though the implementation of early Concat operations was simple, the fixed weighting strategy had difficulties adapting to the complex industrial situation with a variety of defect magnitudes and a high inter-class similarity. PANet improved feature propagation performance by providing the bidirectional paths; however, it did not consider the issue of rational distribution of feature weights. The adaptive paradigm of feature fusion represented by the learnable weighting mechanism of BiFPN moved feature fusion to a dynamically adaptive mechanism that could vary fusion strategies as data characteristics change. However, the current research still faces many challenges: how to achieve more refined feature selection under the premise of maintaining computational efficiency, how to design a more flexible fusion topology to adapt to different defect distribution characteristics, and how to establish a more effective interaction mechanism between global semantic information and local detail information. Solving these problems will further promote the development of steel defect detection technology to higher accuracy and stronger robustness.

## 3. The Proposed MSFE-YOLO Algorithm

**Figure 1:**
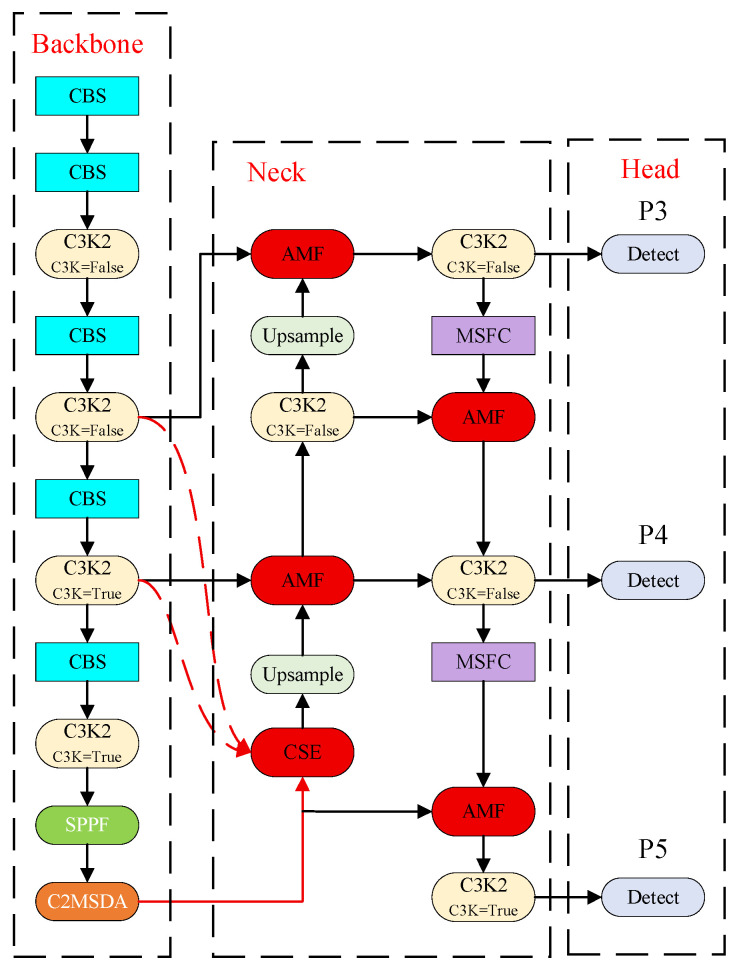
Multi-Scale Frequency-Enhanced YOLO network architecture diagram.

To deal with the issues of the steel surface defect detection tasks, such as the impossibility of detecting multi-scale features with fixed receptive fields, the lack of multi-scale features in form of defect directionality and frequency domain features, and the simple ways of combining features, this paper proposed a steel surface defect detection algorithm named Multi-Scale Frequency-Enhanced YOLO (MSFE-YOLO) that was based on multi-scale frequency domain and defect-aware attention. YOLOv11s was selected as the baseline model because, compared with other recent YOLO variants, it achieved the best comprehensive trade-off among parameter count, computational cost, and detection accuracy across both experimental datasets. By deeply fusing multi-scale, multi-directional, and multi-frequency features, the proposed algorithm achieved high-precision real-time detection. The network architecture was illustrated in Figure 1. The MSFC module was constructed in the neck network. In this module, depth-adaptive dilated convolutions were employed for parallel multi-scale feature extraction, a Laplacian operator was incorporated [46] to explicitly model high-frequency edge information, and an adaptive fusion strategy with learnable weights was utilized, enabling efficient extraction and enhancement of defects across varying scales and fine textures. The C2PSA module in the original YOLOv11s was replaced with the C2MSDA module. By integrating a Sobel operator-based edge-aware module [47], a multi-scale spatial attention mechanism and an adaptive gated fusion strategy, synergistic enhancement of spatial, channel, and edge-domain features were achieved. The AFFE module was developed in the feature fusion stage. Through the introduction of a data-driven weight generation network, a cross-scale feature interaction mechanism and a lightweight fusion strategy, adaptive aggregation of multi-scale features, and cross-level information exchange were enabled.

### 3.1. Multi-Scale Frequency Domain Enhanced Convolution Module

Conventional convolution operations employ fixed-size convolution kernels, whose receptive fields are constrained by kernel size, making it difficult to simultaneously capture defect features at different scales [48]. However, in the actual industrial scene, steel surface defects have the characteristics of scale diversity. The size of tiny pitting defects may be only a few pixels, while large-area patch defects may occupy a significant area of the image. Conventional convolution often needs to expand the receptive field by stacking multilayer networks when dealing with such targets with significant scale differences, but this method not only increases the model depth and computational complexity but also may lead to the disappearance of small-scale defect features in the deep network. In addition, the key features of steel surface defects are often reflected in the changes in edge contour and texture, which are represented as high-frequency components in the frequency domain [49]. Conventional convolution lacks the ability to explicitly model the frequency domain information, resulting in limited ability to extract the defect boundaries and fine textures. At the same time, in the complex industrial background, defect features and background noise are often mixed together. How to adaptively strengthen effective features and suppress redundant information has become the key problem to improve the detection accuracy. Therefore, a Multi-Scale Frequency-Enhanced Convolution module (MSFC) was proposed in this paper. Through multi-scale parallel hole convolution, frequency domain high-frequency enhancement and adaptive dynamic fusion, the defect features of different scales were extracted and enhanced efficiently.

**Figure 2:**
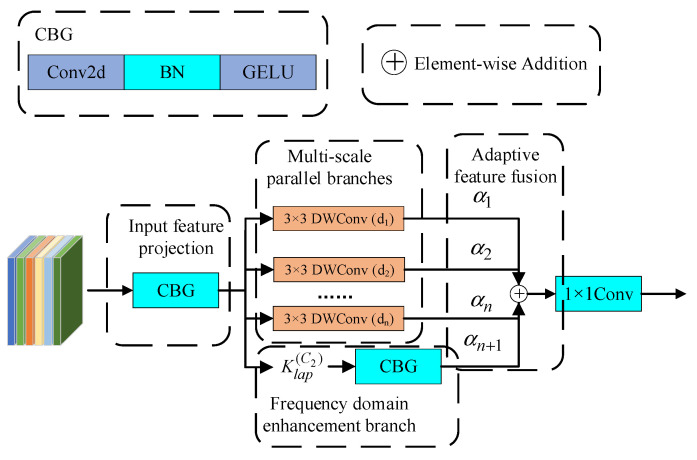
Multi-Scale Frequency-Enhanced Convolution module network architecture diagram.

The network architecture of the MSFC module was shown in Figure 2, including four main components: input feature projection, multi-scale parallel branching, frequency domain enhanced branching, and adaptive feature fusion. First, the input features were adjusted through 1 × 1 convolution. This process could be expressed as follows:

(1) $$X_{proj}=Conv_{1\times 1}(X_{in})$$

where $X_{in}\in {\mathbb{R}}^{C_{1}\times H\times W}$ and $X_{proj}\in {\mathbb{R}}^{C_{2}\times H\times W}$ denote the input and output feature map. $Conv_{1\times 1}$ represents a 1 × 1 convolution operation.

In the multi-scale parallel branches, the module employed depthwise separable convolutions with different dilation rates to extract multi-scale features in parallel. For the *i*-th branch with dilation rate di, the feature extraction process could be expressed as:

(2) $$X_{i}^{dw}=DWConv_{3\times 3,d_{i}}(X_{proj})$$

where $DWConv_{3\times 3,d_{i}}$ denotes a depthwise separable convolution, with a dilation rate of di and a 3 × 3 kernel.

The MSFC module adaptively adjusted the dilation rate configuration according to the network depth. In the shallow network, the smaller dilation rate combination $\left\{1,2,3\right\}$ was used, focusing on local details and small-scale defects. In the middle layer network, the medium dilation rate $\left\{1,2,4,6\right\}$ was adopted, taking into account local characteristics and global context. In the deep network, the larger dilation rate $\left\{1,3,5,7\right\}$ was used to capture the global semantic information. This deep adaptive strategy enabled the MSFC module to extract the most suitable scale features at different stages of the network.

The frequency domain enhancement branch explicitly modeled high-frequency information to strengthen the extraction of defect edges and textures, thereby improving the module’s sensitivity to subtle defects. Together with the multi-scale parallel branches, it established a synergistic relationship between the frequency domain and the spatial domain. Accordingly, this branch was required to respond to high-frequency variations in all orientations of the feature map. The Laplace operator is a second-order differential operator that produces strong responses in regions where the gray-level changes abruptly, such as edges and textures. Because of its isotropic nature, the Laplacian responds equally to rapid intensity changes in every direction, enabling it to capture the overall distribution of high-frequency energy across the feature map without directional bias. This property prevents defect textures oriented in particular directions from being overlooked. Its discrete convolution kernel was defined as:

(3) $$K_{lap}=\left[\begin{array}{ccc} 0 & -1 & 0\\ -1 & 4 & -1\\ 0 & -1 & 0 \end{array}\right]$$

The Laplace operator was used to perform the grouping convolution operation on the projected feature map to extract the high-frequency components of each channel, and then the channel dimension adjustment and information interaction were performed through 1 × 1 convolution:

(4) $$X_{freq}=Conv_{1\times 1}(K_{lap}^{\left(C_{2}\right)}X_{proj})$$

where $K_{lap}^{\left(C_{2}\right)}\in {\mathbb{R}}^{C_{2}\times 1\times 3\times 3}$ indicates that the Laplacian kernel was copied *C*_2_ times in the channel dimension.

The adaptive dynamic fusion mechanism realized the adaptive aggregation of multi-branch features through the learnable weight parameters. Let the total number of branches be *N*, including *N* − 1 multi-scale branch and one frequency domain branch. The fusion process was defined as:

(5) $$X_{fuse}=\sum_{i=1}^{N}\alpha_{i}X_{i}$$

where $X_{i}$ denotes the output feature map of the *i*-th branch, and $\alpha_{i}$ represents the corresponding fusion weight:

(6) $$\alpha_{i}=\frac{\exp(w_{i})}{\sum_{j=1}^{N}\exp(w_{i})}$$

where $w_{i}$ denotes the learnable fusion parameter.

The fused features underwent final feature transformation through the output projection layer:

(7) $$X_{out}=Conv_{1\times 1}(X_{fuse})$$

### 3.2. Multi-Scale Defect-Aware Attention Module

**Figure 3:**
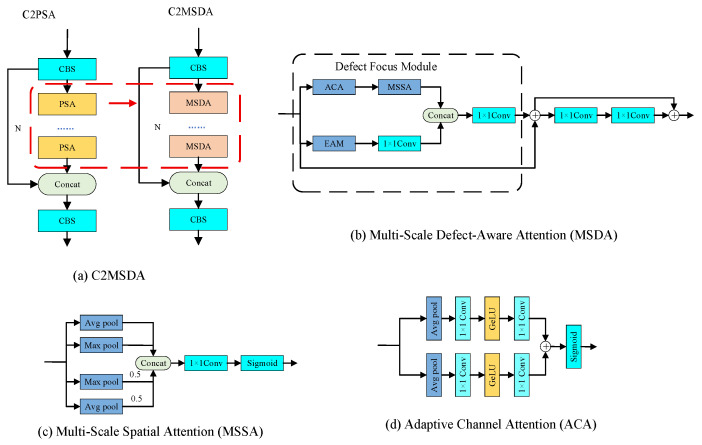
Cross-Stage Partial with Multi-Scale Defect-Aware Attention module network architecture diagram.

Although the C2PSA module of the original YOLOv11 algorithm showed good performance in feature extraction, it still had obvious limitations in industrial scenes such as steel surface defect detection. Firstly, the position-sensitive attention mechanism adopted by C2PSA mainly focused on the global relationship modeling of different positions in the feature map, but the ability to capture local details such as defect edges and shapes was insufficient. Secondly, the attention calculation process of this module lacked explicit modeling of multi-scale information, which was unable to effectively deal with the characteristics of large-scale changes and diverse forms of steel surface defects. In addition, the feature fusion strategy of C2PSA was relatively simple, and it failed to make full use of the complementarity of multi-dimensional information such as spatial domain, channel domain, and edge domain, resulting in the limited detection accuracy of small-size defects and low-contrast defects. Therefore, this paper replaced the PSA module in the C2PSA module with the MSDA module to construct the C2MSDA module. By introducing the mechanisms of edge perception, multi-scale spatial attention, and adaptive channel attention, a more refined learning framework for defect feature representation was constructed. The network architecture of the MSDA module was shown in Figure 3. It was composed of the Defect Focus Module (DFM), which was internally integrated with edge-aware module (EAM), Multi-Scale Spatial Attention Module (MSSA), and Adaptive Channel Attention Module (ACA).

**Figure 4:**
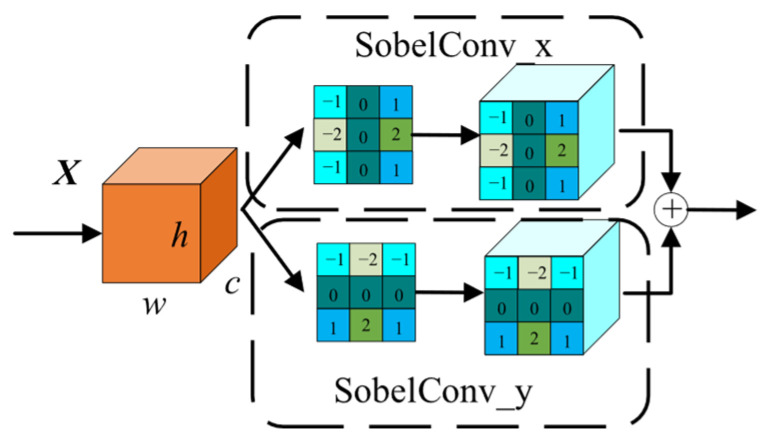
Edge-aware module network architecture diagram.

The EAM sub-module provided explicit edge priors for the Defect Focus Module (DFM), guiding attention toward defect boundary regions. Unlike the frequency domain enhancement branch in MSFC, the EAM was not concerned with the overall distribution of high-frequency energy but rather with the orientation and gradient structure of edges. Common steel surface defects such as cracks and scratches typically manifest as abrupt gray-level transitions extending along specific directions, exhibiting pronounced directional characteristics. Such defect properties required the EAM to perceive gradient variations along different orientations separately. The network architecture of the EAM was illustrated in Figure 4. By introducing the Sobel operator, the module explicitly extracted edge gradient information from the feature maps to enhance boundary perception. The detailed workflow was as follows:

For the input feature map $X_{in}\in {\mathbb{R}}^{C_{1}\times H\times W}$, Sobel convolution kernels in horizontal and vertical directions were applied for edge detection. The horizontal Sobel operator was defined as:

(8) $$G_{x}=\left[\begin{array}{ccc} -1 & 0 & 1\\ -2 & 0 & 2\\ -1 & 0 & 1 \end{array}\right]$$

The vertical Sobel operator was defined as:

(9) $$G_{y}=\left[\begin{array}{ccc} -1 & -2 & -1\\ 0 & 0 & 0\\ 1 & 2 & 1 \end{array}\right]$$

The gradient responses in horizontal and vertical directions were computed via depthwise convolution for each channel:

(10) $$X_{x}=DConv_{G_{x}}(X)$$

(11) $$X_{y}=DConv_{G_{y}}(X)$$

where DConv denotes the depthwise convolution operation.

The edge intensity map was calculated using gradient magnitude:

(12) $$X_{mag}=\sqrt{X_{x}^{2}+X_{y}^{2}+\varepsilon }$$

where $\varepsilon =10^{-6}$ is a numerical stability term.

Finally, the edge features were semantically enhanced through 1 × 1 convolution:

(13) $$X_{eam}=Conv_{1\times 1}(X_{mag})$$

The network architecture of the MSSA sub-module was illustrated in Figure 3c. By capturing spatial saliency information at different scales, the adaptability of the network to multi-scale defects was enhanced. The workflow was as follows:

First, max pooling and average pooling along the channel dimension were computed to generate spatial descriptors:

(14) $$X_{\max}=MAX(X)$$

(15) $$X_{avg}=GAP(X)$$

where MAX(•) and GAP(•) denote max pooling and average pooling, respectively.

To incorporate multi-scale information, pooling features with attenuation weights were constructed from the obtained spatial descriptors:

(16) $$X_{\max}^{\prime }=0.5X_{\max}$$

(17) $$X_{avg}^{\prime }=0.5X_{avg}$$

The four spatial feature maps were concatenated along the channel dimension to obtain multi-scale spatial features:

(18) $$X_{cat}=Concat(X_{\max},X_{avg},X_{\max}^{\prime },X_{avg}^{\prime })$$

where Concat(•) denotes the concatenation operation along the channel dimension.

Finally, the spatial attention map was generated through 7 × 7 convolution and the Sigmoid activation function:

(19) $$X_{mssa}=\sigma (Conv_{7\times 7}(X_{cat}))$$

where $Conv_{7\times 7}$ represents the 7 × 7 convolution operation, and σ denotes the Sigmoid function.

The network architecture of the ACA sub-module was shown in Figure 3d. By modeling the inter-channel dependencies, the channel feature responses were adaptively recalibrated to highlight discriminative channels relevant to defects. The workflow was as follows:

First, global average pooling and global max pooling were employed to compress the spatial dimension information. The channel attention weights were then learned through a shared multilayer perceptron (MLP), with the reduction ratio set to 16:

(20) $$W_{avg}=MLP(F_{avg})$$

(21) $$W_{\max}=MLP(F_{\max})$$

where MLP(•) represents the multilayer perceptron consisting of two 1 × 1 convolutional layers. $F_{avg}$ and $F_{\max}$ denote the channel-wise average-pooled and max-pooled feature maps, respectively.

Finally, the channel attention map was generated by fusing the outputs of the two branches with the Sigmoid activation function:

(22) $$X_{aca}=\sigma (W_{avg}+W_{\max})$$

The DFM synergistically integrated the multi-dimensional features produced by the above three sub-modules and achieved dynamic feature fusion through an adaptive gating mechanism. The module first applied channel attention followed by spatial attention to the input features *X* to obtain enhanced features:

(23) $$X_{attn}=X⊙X_{aca}⊙X_{mssa}$$

where ⊙ denotes element-wise multiplication.

The edge features $X_{eam}$ output by the EAM sub-module generated adaptive weights through the gating network:

(24) $$W_{edge}=\sigma (Conv_{1\times 1}(X_{eam}))$$

Therefore, the edge-enhanced features were:

(25) $$X_{edge}=X⊙W_{edge}$$

The spatial-channel enhanced features and edge-enhanced features were concatenated along the channel dimension and then mapped back to the original channel dimension through 1 × 1 convolution:

(26) $$X_{fused}=Conv_{1\times 1}(Concat(X_{attn},X_{edge}))$$

The MSDA module employed a residual connection strategy, adding the output of the a DFM to the input features:

(27) $$X_{out}^{1}=X+X_{fused}$$

To further enhance the feature representation capability, a convolutional feedforward layer was introduced for nonlinear transformation, which consisted of two 1 × 1 convolutions:

(28) $$X_{ffn}=Conv_{1\times 1}(Conv_{1\times 1}(X_{out}^{1}))$$

Therefore, the final output of the MSDA module was:

(29) $$X_{out}=X_{ffn}+X_{out}^{1}$$

### 3.3. Adaptive Feature Fusion Enhancement Module

**Figure 5:**
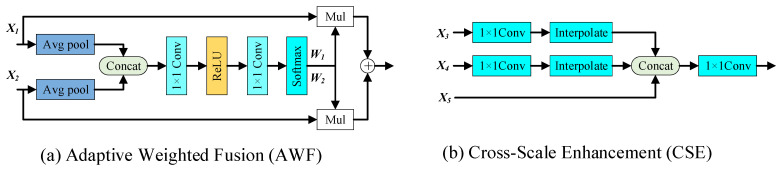
Adaptive Feature Fusion Enhancement module network architecture diagram.

Traditional detection algorithms such as YOLOv11 typically employ simple channel concatenation operations to fuse features from different network layers, where multiple feature maps are directly concatenated along the channel dimension to form higher-dimensional feature representations. The simple concatenation operation assigns the same importance to all input features, ignoring the differential contribution of different feature layers in representing different scale targets, resulting in high redundancy and insufficient discrimination of the fused feature representation. Features from different depths have different semantic levels and spatial resolutions. Shallow features contain rich spatial details, but semantic information is weak. Deep features have strong semantic information, but spatial details are lost. Direct concatenation operation was unable to make full use of this complementarity. To address these limitations, this paper proposed an Adaptive Feature Fusion Enhancement (AFFE) module, comprising an Adaptive Weighted Fusion (AWF) sub-module and a Cross-Scale Enhancement (CSE) sub-module, as illustrated in Figure 5. Compared with the traditional feature fusion method, the AFFE module realized the adaptive feature fusion through the data-driven weight generation network, which could dynamically adjust the contribution of different features according to the task requirements and avoid the information redundancy caused by the fixed weight. The AFFE module could explicitly model the cross-scale feature interaction, so that the deep features could directly obtain the details of the shallow layer and enhance the detection ability of small-scale defects.

The network architecture of the AWF sub-module was illustrated in Figure 5a. The AWF module employed a lightweight attention network to assign adaptive weights to features from different sources, enabling the network to dynamically adjust the fusion strategy based on the semantic content and spatial distribution of input features. The workflow was described as follows:

Given *N* input feature maps $X_{i}\in {\mathbb{R}}^{C_{i}\times H\times W}$ to be fused, global average pooling was first applied to each feature map to extract global contextual information:

(30) $$G_{i}=GAP(X_{i})$$

The global descriptors of all features were concatenated along the channel dimension to obtain an aggregated global feature representation:

(31) $$G_{cat}=Concat(G_{1},\ldots ,G_{i},\ldots ,G_{n})$$

The fusion weights for *N* features were learned through a multilayer perceptron (MLP) and normalized using the Softmax activation function:

(32) $$W_{i}=Soft\max(MLP(G_{cat}))$$

where Softmax(•) denotes the Softmax activation function, as defined in Equation (6). The MLP consisted of two 1 × 1 convolutional layers with a ReLU activation function. The first layer compressed the concatenated global features from *C* dimensions to *C*/8 dimensions. After ReLU activation, the second layer mapped the hidden representation back to the original *C*-dimensional vector, which was then normalized by the Softmax function to produce the fusion weights for each feature level.

The feature maps of different dimensions were then combined through weighted summation:

(33) $$X_{awf}=\sum_{i=1}^{N}W_{i}X_{i}$$

The network architecture of the CSE sub-module was depicted in Figure 5b. The CSE module enhanced the representational capacity of the deepest feature map through cross-level information interaction, enabling it to incorporate fine-grained details from shallow features while maintaining a large receptive field. The workflow proceeded as follows:

The CSE sub-module received feature maps ($X_{3}\in {\mathbb{R}}^{C_{3}\times H_{3}\times W_{3}}$, $X_{4}\in {\mathbb{R}}^{C_{4}\times H_{4}\times W_{4}}$, and $X_{5}\in {\mathbb{R}}^{C_{5}\times H_{5}\times W_{5}}$) from three different levels of the feature pyramid as input, where subscripts 3, 4, and 5 denote the three pyramid levels, typically satisfying $H_{3}>H_{4}>H_{5}$ and $W_{3}>W_{4}>W_{5}$.

To achieve cross-scale information transmission, the CSE sub-module first performed channel mapping on features at different levels through 1 × 1 convolution:

(34) $$T_{3\to 5}=Conv_{1\times 1}^{\left(3\to 5\right)}(X_{3})$$

(35) $$T_{4\to 5}=Conv_{1\times 1}^{\left(4\to 5\right)}(X_{4})$$

where $Conv_{1\times 1}^{\left(3\to 5\right)}$ denotes a 1 × 1 convolution operation with C3 input channels and C5 output channels.

The feature maps were downsampled to match the spatial dimensions of X_5_ via bilinear interpolation:

(36) $${\tilde{T}}_{3\to 5}=Interpolate(T_{3\to 5},(H_{5},W_{5}))$$

(37) $${\tilde{T}}_{4\to 5}=Interpolate(T_{4\to 5},(H_{4},W_{5}))$$

where Interpolate(•) denotes the bilinear interpolation operation.

The original X_5_ feature was concatenated with the projected features from other levels along the channel dimension:

(38) $$X_{5}^{cat}=Concat(X_{5},{\tilde{T}}_{3\to 5},{\tilde{T}}_{4\to 5})$$

Finally, the multi-scale features were mapped back to the original channel dimension through 1 × 1 convolution:

(39) $$X_{5}^{enhanced}=Conv_{1\times 1}(X_{5}^{cat})$$

## 4. Results and Analysis

### 4.1. Experimental Platform

All experiments in this paper were trained and tested on the same server. The server GPU was equipped with an NVIDIA GeForce RTX 3090, 24 g video memory (NVIDIA Corporation, Santa Clara, CA, USA) and the CPU was Intel Xeon e5-2680 v4@2.40 GHz (Intel Corporation, Santa Clara, CA, USA). The operating system was Ubuntu20.04, the deep learning framework was PyTorch 2.0.0, and the programming language was Python 3.8. In addition, to verify the deployment capability on edge devices, inference speed tests were conducted on the NVIDIA Jetson AGX Xavier platform. This platform is equipped with a 512-core Volta GPU and 32 GB of shared memory, running the JetPack 5.1 environment, and is a commonly used edge computing device in industrial settings. All models were tested with the same inference settings as on the server side.

### 4.2. Datasets

This paper used NEU-DET [50] and GC10-DET [2] to verify the effectiveness of the proposed algorithm. The NEU-DET dataset was collected by Northeastern University and contained 1800 gray-scale images with a size of 200 × 200 pixels, covering six typical hot-rolled steel strip surface defect categories: crazing, inclusion, patches, pitted surface, rolled-in scale, and scratches. In this paper, NEU-DET dataset was divided into training set, verification set, and test set according to a 7:1:2 ratio. The GC10-DET dataset was collected from real industrial scenes and contained 2294 images with a size of 2048 × 1000 pixels, covering 10 steel surface defect categories: punching hole, welding line, crescent gap, water spot, oil spot, silk spot, inclusion, rolled pit, crease, and waist folding. The GC10-DET dataset was also divided into training set, verification set and test set according to the 7:1:2 ratio. The diversity of defect types across the two datasets, ranging from texture-based defects such as crazing and scratches to shape-based defects such as crescent gap and waist folding, provides a comprehensive evaluation of the proposed algorithm’s generalization capability under varying defect morphologies and imaging conditions.

### 4.3. Experimental Settings

All test parameters in this paper were basically set according to the parameter configuration recommended by YOLOv11. Two data enhancement methods, Mosaic [51] and MixUp [52] were used in the data preprocessing stage, and Mosaic enhancement was turned off in the last 10 epochs of training. The HSV color–space jittering was applied to the training images, with the hue shift coefficient set to 0.015, the saturation shift coefficient set to 0.7, and the value (brightness) shift coefficient set to 0.4. The probability of random horizontal flipping was set to 0.5, the random translation fraction was set to 0.1, and the random scaling fraction was set to 0.5. The input image was uniformly adjusted to the 640 × 640 size, and the adaptive anchor box calculation strategy was adopted. In the training phase, the stochastic gradient descent (SGD) optimizer was used to update the network parameters. The initial learning rate was set to 0.01, the learning rate momentum was 0.937, and the weight attenuation coefficient was 0.0005. The batch size was set to 16, the number of CPU threads was 8, and the number of training rounds (epoch) was 300.

### 4.4. Evaluation Metrics

In this paper, multiple metrics were used to comprehensively evaluate the performance of the model. Precision (P) represents the proportion of correctly detected targets in the predicted positive samples:

(40) $$P=\frac{TP}{TP+FP}\times 100\%$$

Recall (R) represents the proportion of actually detected targets relative to all ground-truth targets:

(41) $$R=\frac{TP}{TP+FN}\times 100\%$$

where TP (true positive) denotes the number of correctly predicted positive samples, FP (false positive) denotes the number of negative samples incorrectly predicted as positive, and FN (false negative) denotes the number of positive samples incorrectly predicted as negative.

Average precision (AP) is calculated as the area under the precision–recall curve:

(42) $$AP=\int_{0}^{1}P(R)dR$$

Mean average precision (mAP) is computed as the average of AP values across all categories:

(43) $$mAP=\frac{1}{n}\sum_{i=1}^{n}AP(i)\times 100\%$$

where n denotes the total number of categories.

The F_1_ score provides a harmonic mean of precision and recall:

(44) $$F_{1}=\frac{2P\times R}{P+R}$$

Additionally, the parameters, GFLOPs, and FPS (frames per second) were employed to evaluate model complexity and real-time performance.

### 4.5. Experimental Results and Analysis

#### 4.5.1. Result Analysis of the MSFC Module

To verify the effectiveness of the proposed MSFC module in defect detection, the conventional convolutions in the neck network of the baseline YOLOv11s model were replaced with the MSFC, DynamicConv [53], DySnakeConv [21], LDConv [54], and MSCB [55] modules, respectively. The experimental results are presented in Table 1.

On the NEU-DET dataset, comparative experiments were conducted by replacing the conventional convolutions in the YOLOv11s neck network with five different convolution modules: MSFC, DynamicConv, DySnakeConv, LDConv, and MSCB. The experimental results demonstrated that the model equipped with the proposed MSFC module achieved a precision of 75.9%, representing an improvement of 6.1% over the baseline YOLOv11s. The F1 score reached 75.4%, which is 2.9% higher than the baseline, and mAP@0.5 reached 78.7%, an improvement of 0.6%. In terms of model complexity, the MSFC module had 8.92 M parameters, slightly lower than the baseline model’s 8.99 M, with a computational cost of 23.6 GFLOPs and an inference speed of 109.9 FPS. In contrast, although DySnakeConv achieved an mAP@0.5 of 78.5%, it required 12.08 M parameters and 30.0 GFLOPs, with an inference speed of only 70.9 FPS. Both DynamicConv and MSCB modules yielded an mAP@0.5 of 77.5%, which was lower than the baseline model, indicating that these two convolution structures were not suitable for steel surface defect detection tasks.

The multi-scale parallel dilated convolution branch, through its depth-adaptive dilation rate configuration, could simultaneously capture defect features at different scales, effectively addressing the challenge of large-scale variations in steel surface defects. This enabled the model to achieve strong detection capability for both small-scale pitting defects and large-area patch defects. The frequency domain enhancement branch explicitly modeled high-frequency edge information by incorporating the Laplacian operator, strengthening the network’s perception of defect contours and texture variations and compensating for the limitations of conventional convolutions in frequency domain feature extraction. This was particularly effective for scratches and cracks with prominent edge features. The adaptive dynamic fusion mechanism performed weighted aggregation of multi-branch features through learnable weight parameters, enabling the network to automatically adjust the contribution ratio of each branch according to the characteristics of the input data. This approach avoided the information redundancy problem caused by fixed weight fusion strategies while maintaining low computational overhead and improving detection accuracy.

#### 4.5.2. Result Analysis of the AWF Module

To verify the effectiveness of the proposed Adaptive Weighted Fusion (AWF) sub-module in feature fusion, comparative experiments were conducted by replacing the Concat operation in the neck network of the baseline YOLOv11s model with the BiFPN module [42] and the AWF module, respectively. The experimental results were presented in Table 2. The results showed that the model with the AWF module achieved a precision of 73.3%, which is 3.5% higher than the baseline YOLOv11s, and an mAP@0.5 of 78.5%, representing an improvement of 0.4% over the baseline. In terms of model complexity, the AWF module had 10.32 M parameters and a computational cost of 23.0 GFLOPs, with an inference speed of 109.9 FPS, which satisfied the requirements for real-time detection. In contrast, although the BiFPN module maintained the same parameter count and computational cost as the baseline model, its mAP@0.5 was only 78.4%, showing a slightly lower improvement compared to the AWF module.

BiFPN achieved multi-scale feature fusion through a bidirectional feature pyramid structure with simple learnable weights, where each input feature was weighted by only a single scalar parameter. In contrast, the proposed AWF module adopted a more refined weight generation strategy. It first extracted global contextual information from each feature layer through global average pooling, then employed a multilayer perceptron (MLP) to learn complex dependencies among features, and finally generated adaptive fusion weights through Softmax normalization. This data-driven weight generation mechanism could dynamically adjust the contribution ratio of each feature layer based on the semantic content and spatial distribution of input features, resulting in more discriminative fused feature representations.

#### 4.5.3. Ablation Experiment

To verify the contribution of each proposed module to the detection performance, ablation experiments were conducted on the NEU-DET dataset, and the results were presented in Table 3. The baseline YOLOv11s model had 8.99 M parameters, a computational cost of 21.6 GFLOPs, an mAP@0.5 of 78.1%, an mAP@0.5:0.95 of 46.2%, and an inference speed of 137.0 FPS. When the MSFC module was added individually, mAP@0.5 increased to 78.7%, which represents an improvement of 0.6%. When the C2MSDA module was added individually, mAP@0.5 increased to 78.5%, which represents an improvement of 0.4%. When the AFFE module was added individually, mAP@0.5 increased to 78.8%, which represents an improvement of 0.7%. When the MSFC and C2MSDA modules were added simultaneously, the model achieved an mAP@0.5 of 79.2%, representing an improvement of 1.1% over the baseline while maintaining the same parameter count of 8.99 M. When all three modules were incorporated, the MSFE-YOLO model achieved an mAP@0.5 of 79.8%, which is an improvement of 1.7% over the baseline, with an mAP@0.5:0.95 of 45.8%, 11.69 M parameters, a computational cost of 27.9 GFLOPs, and an inference speed of 89.3 FPS, which satisfied the real-time detection requirements in industrial scenarios. To further validate the feasibility of edge deployment, the inference speed of each configuration was tested on the Jetson AGX Xavier platform (last column of Table 3). The baseline YOLOv11s achieves 32.3 FPS, while MSFE-YOLO achieves 22.1 FPS, a decrease of approximately 31.6%, which is consistent with the reduction ratio observed on the RTX 3090. In practical hot-rolling production lines, the frame rate requirement for quality inspection typically does not exceed 15 FPS, so 22.1 FPS fully satisfies the real-time detection requirement in industrial scenarios. Moreover, the current tests are based on direct PyTorch inference, and further acceleration is achievable in actual deployment through optimization techniques such as TensorRT.

The MSFC module extracts rich multi-scale features through multi-scale parallel dilated convolutions and the frequency domain enhancement mechanism, while the C2MSDA module performs refined feature enhancement through edge perception, multi-scale spatial attention, and adaptive channel attention. These two modules complement each other at the feature extraction and feature enhancement levels, jointly improving the model’s detection capability for different types of defects. The AFFE module further optimizes the aggregation strategy of multi-scale features through Adaptive Weighted Fusion and cross-scale feature enhancement mechanisms, enabling deep semantic information and shallow detail information to fully interact and integrate. This further improved the detection accuracy of the model building upon the MSFC and C2MSDA modules. The three modules collaboratively optimize network performance from three dimensions: feature extraction, feature enhancement, and feature fusion. The ablation experiment results fully validate the effectiveness and necessity of each module design proposed in this paper.

#### 4.5.4. Comparative Experiment Analysis

To verify the effectiveness of the proposed algorithm, MSFE-YOLO was compared with Faster R-CNN [56], SSD [57], YOLOv5s, YOLOv8s, YOLOv9s [58], YOLOv10s [59], and YOLOv11s on the NEU-DET and GC10-DET public datasets. The experimental results are presented in Table 4 and Table 5, respectively.

On the NEU-DET dataset, the proposed MSFE-YOLO algorithm achieved the best detection performance. As shown in Table 4, MSFE-YOLO achieved an mAP@0.5 of 79.8%, an improvement of 1.7% over the baseline YOLOv11s model. The precision reached 78.9%, which is an improvement of 9.1%, and the F1 score was 75.6%, which is an improvement of 3.1%. Compared with other algorithms, MSFE-YOLO outperformed Faster R-CNN, SSD, YOLOv5s, YOLOv8s, YOLOv9s, and YOLOv10s in mAP@0.5 by 24.7%, 33.8%, 2.5%, 1.0%, 3.4%, and 1.7%, respectively. Although the two-stage detection algorithm Faster R-CNN achieved a recall of 81.6%, its precision was only 19.1%, resulting in an mAP@0.5 of only 55.1%. The SSD algorithm had relatively high precision but extremely low recall, leading to unsatisfactory overall performance. In terms of model complexity, MSFE-YOLO had 11.69 M parameters and a computational cost of 27.9 GFLOPs. Compared with Faster R-CNN, which had 136.79 M parameters and 369.8 GFLOPs, MSFE-YOLO achieved significant model lightweighting while substantially improving detection accuracy.

On the GC10-DET dataset, the proposed algorithm also demonstrated good detection performance and generalization capability. As shown in Table 5, MSFE-YOLO achieved an mAP@0.5 of 66.7%, which is an improvement of 2.1% over the baseline YOLOv11s model, and an mAP@0.5:0.95 of 34.4%, which is an improvement of 2.4%. Compared with YOLOv5s, YOLOv8s YOLOv9s, and YOLOv10s, MSFE-YOLO improved mAP@0.5 by 3.1%, 2.5%, 2.5%, and 7.4%, respectively. Faster R-CNN and SSD performed poorly on this dataset, with mAP@0.5 of only 35.3% and 39.2%, respectively, struggling to handle the characteristics of the GC10-DET dataset, which exhibits high inter-class similarity and imbalanced sample distribution. MSFE-YOLO achieved a recall of 61.6%, which is an improvement of 2.1% over YOLOv11s’s 59.5%, indicating that the proposed algorithm provides better suppression of missed detections. Additionally, the mAP@0.5:0.95 of all compared algorithms on the GC10-DET dataset remained at a relatively low level, which was primarily attributed to the inherent characteristics of the dataset: the original image resolution was 2048 × 1000, and scaling to the 640 × 640 input size introduced significant geometric distortion; the 10 defect categories exhibited large morphological differences, and horizontal rectangular bounding boxes had limited descriptive capability for irregular defects such as crescent gaps and waist folding, which increased the matching difficulty under high IoU thresholds. Nevertheless, MSFE-YOLO achieved an mAP@0.5:0.95 of 34.4%, which was the highest among all compared methods and represented a 2.4% improvement over the baseline.

The GC10-DET dataset contained 10 defect categories, with some images exhibiting multiple coexisting defect types, which imposed higher requirements on feature extraction and fusion capabilities. The frequency domain enhancement mechanism of the MSFC module could effectively distinguish defects with similar textures but different categories, such as water spots and oil spots. The multi-scale dilated convolution branch enhanced adaptability to defects with large-scale variations, such as punching holes and rolled pits. The adaptive gated fusion strategy of the C2MSDA module dynamically adjusted the contribution weights of each attention branch based on input features, effectively addressing the feature confusion problem caused by high inter-class similarity. The cross-scale feature enhancement mechanism of the AFFE module facilitated information interaction among features at different levels, enabling the model to balance global semantic understanding and local detail localization when handling scenarios with multiple coexisting defects, thereby achieving stable and reliable detection performance in complex industrial scenarios.

**Figure 6:**
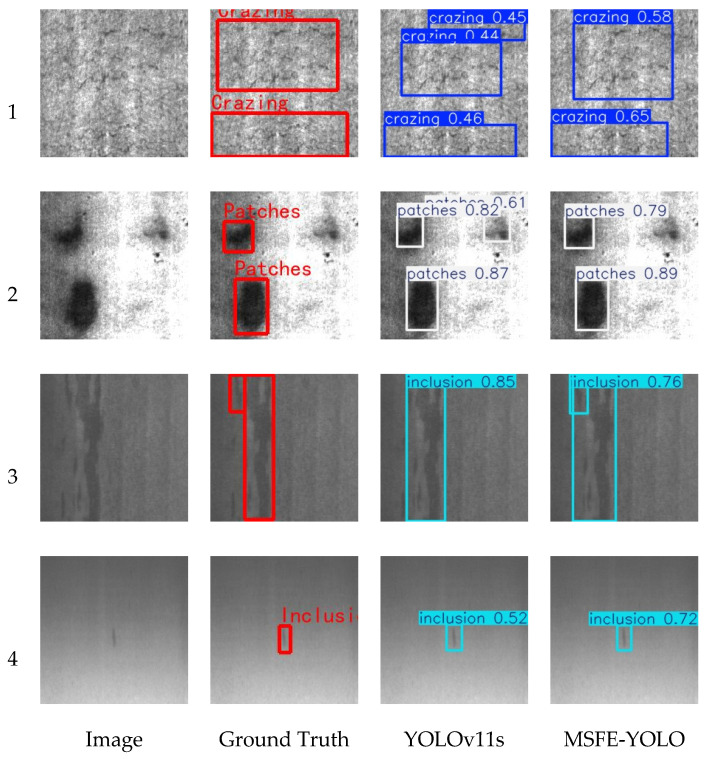
Visualization results on the NEU-DET dataset.

**Figure 7:**
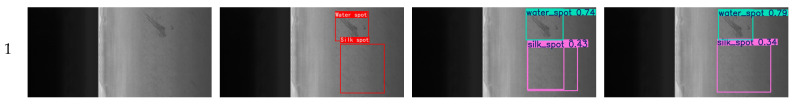

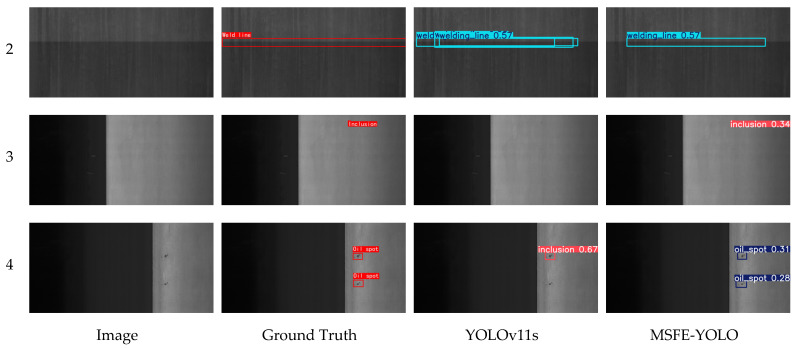
Visualization results on the GC10-DET dataset.

To further visually demonstrate the detection effectiveness of the proposed algorithm, typical samples were selected from the NEU-DET and GC10-DET datasets for visual comparative analysis. The results are shown in Figure 6 and Figure 7.

In the visualization results on the NEU-DET dataset, MSFE-YOLO demonstrated superior detection performance compared to YOLOv11s. As shown in rows 1 and 2 of Figure 6, YOLOv11s exhibited false positives in complex background regions, misidentifying some background textures as defect targets, whereas MSFE-YOLO accurately identified the actual defect regions without generating false positives. As shown in row 3 of Figure 6, YOLOv11s missed some low-contrast and small-sized defects, while MSFE-YOLO successfully detected these defect targets. Furthermore, as shown in row 4 of Figure 6, for the same defect targets, the confidence scores output by MSFE-YOLO were significantly higher than those of YOLOv11s, indicating that the proposed algorithm possesses stronger discriminative capability for defect features.

In the visualization results on the GC10-DET dataset, MSFE-YOLO also demonstrated clear advantages. As shown in rows 1 and 2 of Figure 7, YOLOv11s exhibits over-detection during the detection process, generating multiple redundant responses for defect regions, whereas MSFE-YOLO does not exhibit such issues. As shown in rows 3 and 4 of Figure 7, YOLOv11s suffered from missed detections, with some defect targets remaining undetected, while MSFE-YOLO achieved complete detection. Notably, row 4 of Figure 7 also reveals that YOLOv11s produced misclassifications, incorrectly identifying certain defect types as other categories, whereas MSFE-YOLO correctly recognized the defect types, demonstrating stronger category discrimination capability.

## 5. Conclusions

To address the challenges of difficult multi-scale feature extraction, insufficient edge information modeling, and low feature fusion efficiency in steel surface defect detection tasks, this paper proposed MSFE-YOLO, a detection algorithm that integrated multi-scale frequency domain enhancement with defect-aware attention. The algorithm achieved significant improvements in detection performance through the collaborative design of three core modules: the Multi-Scale Frequency-Enhanced Convolution module, which employed depthwise separable convolutions with different dilation rates to capture multi-scale spatial features, combined with the Laplacian operator to enhance high-frequency responses at defect edges, effectively overcoming the limitations of conventional convolutions in scale adaptability and frequency domain modeling; the Multi-Scale Defect-Aware Attention module, which achieved multi-dimensional enhancement of defect features by integrating edge perception, spatial attention, and channel attention sub-modules, significantly improving detection accuracy for small-sized and low-contrast defects; and the Adaptive Feature Fusion Enhancement module, which dynamically allocated fusion weights through a lightweight attention network and facilitated information interaction among features at different levels via Cross-Scale Enhancement mechanisms, overcoming the limitation of fixed weights in traditional concatenation operations. Experiments on the NEU-DET and GC10-DET datasets validated the effectiveness of the proposed algorithm. MSFE-YOLO achieved superior detection accuracy compared to existing methods while maintaining high inference speed, and ablation experiments further demonstrated the rationality and necessity for each module design.

Although the proposed algorithm has achieved promising results in steel defect detection tasks, several limitations remain to be addressed. On one hand, the mAP@0.5:0.95 metric on the GC10-DET dataset, despite showing improvement, remains at a relatively low level, indicating that the localization accuracy of the model under high IoU thresholds requires further optimization. On the other hand, the introduction of multiple functional modules increases the model’s parameter count and computational cost, posing certain challenges for deployment on edge devices. Future research will proceed in the following directions: first, to address the limited localization accuracy under high IoU thresholds, future work will explore regression loss functions more suitable for industrial defect detection (e.g., Shape-IoU Loss), investigate adaptive aspect-ratio-preserving preprocessing strategies to reduce the geometric distortion caused by extreme aspect ratios and explore oriented bounding box detection methods to provide more precise localization for irregularly shaped defects; second, to explore more lightweight module designs to reduce model complexity while maintaining detection accuracy; and third, to incorporate knowledge distillation or model pruning techniques to further compress the model for resource-constrained industrial deployment environments; additionally, it could integrate richer data augmentation strategies and semi-supervised learning methods to address the issue of insufficient labeled data in actual production, thereby promoting the migration and application of the algorithm to a broader range of industrial defect detection scenarios.

## Figures and Tables

**Table 1:** MSFC module results.

Method	P	R	F_1_	mAP@0.5	mAP@0.5:0.95	Parameters	GFLOPs	FPS (f/s)
YOLOv11s	69.8	75.5	72.5	78.1	46.2	8.99	21.6	137.0
YOLOv11s-DynamicConv	71.6	75.8	73.6	77.5	45.1	11.10	20.6	122.0
YOLOv11s-DySnakeConv	75.1	73.2	74.1	78.5	45.2	12.08	30.0	70.9
YOLOv11s-LDConv	71.6	72.6	72.1	78.3	45.6	8.54	21.1	95.2
YOLOv11s-MSCB	70.8	74.2	72.5	77.5	44.9	8.95	22.2	128.2
YOLOv11s-MSFC	75.9	75.0	75.4	78.7	44.9	8.92	23.6	109.9

**Table 2:** AWF module results.

Method	P	R	F_1_	mAP@0.5	mAP@0.5:0.95	Parameters	GFLOPs	FPS (f/s)
YOLOv11s	69.8	75.5	72.5	78.1	46.2	8.99	21.6	137.0
YOLOv11s-BiFPN	73.4	73.4	73.4	78.4	45.3	8.99	21.6	129.9
YOLOv11s-AWF	73.3	72.8	73.0	78.5	45.2	10.32	23.0	109.9

**Table 3:** Ablation experiments.

YOLOv11s	MSFC	C2MSDA	AFFE	Parameters	GFLOPs	mAP@0.5/(%)	mAP@0.5:0.95/(%)	FPS (f/s)	
								RTX 3090	Xavier AGX
√				8.99	21.6	78.1	46.2	137.0	32.3
√	√			8.92	23.6	78.7	44.9	109.9	27.4
√		√		9.06	21.6	78.5	45.4	129.9	29.9
√			√	11.70	25.8	78.8	44.9	113.6	27.8
√	√	√		8.99	23.7	79.2	44.5	109.9	26.6
√	√	√	√	11.69	27.9	79.8	45.8	89.3	22.1

√ indicates that the model used includes this module.

**Table 4:** Comparative experiment on the NEU-DET dataset.

Model	Parameters	GFLOPs	P	R	F1	mAP@0.5/(%)	mAP@0.5:0.95/(%)
Faster R-CNN	136.79	369.8	19.1	81.6	31.0	55.1	25.6
SSD	12.34	38.8	78.6	15.8	26.3	46.0	18.8
YOLOv5s	6.71	16.0	73.3	74.5	73.9	77.3	41.2
YOLOv8s	10.62	28.7	76.8	72.7	74.7	78.8	44.9
YOLOv9s	6.83	26.7	73.7	72.7	73.2	78.3	44.6
YOLOv10s	6.89	21.4	72.8	72.1	72.5	76.4	43.8
YOLOv11s	8.99	21.6	69.8	75.5	72.5	78.1	46.2
MSFE-YOLO	11.69	27.9	78.9	72.6	75.6	79.8	45.8

**Table 5:** Comparative experiment on the GC10-DET dataset.

Model	Parameters	GFLOPs	P	R	F1	mAP@0.5/(%)	mAP@0.5:0.95/(%)
Faster R-CNN	136.87	369.9	17.8	46.6	25.8	35.3	16.1
SSD	12.87	40.0	83.8	15.2	25.7	39.2	15.5
YOLOv5s	6.72	16.0	74.3	58.7	65.6	63.6	32.7
YOLOv8s	10.62	28.7	63.7	62.7	63.2	64.2	30.9
YOLOv9s	6.84	26.7	72.6	55.4	62.8	64.2	30.8
YOLOv10s	6.89	21.4	61.8	53.9	57.6	59.3	30.2
YOLOv11s	8.99	21.6	67.8	59.5	63.4	64.6	32.0
MSFE-YOLO	11.69	27.9	64.0	61.6	62.8	66.7	34.4

## Data Availability

The raw data supporting the conclusions of this article will be made available by the authors on request.
